# Coupling coordination degree of healthcare resource supply, demand and elderly population change in China

**DOI:** 10.1186/s12939-024-02236-x

**Published:** 2024-07-25

**Authors:** Mengyuan Ma, Leiyu Shi, Wanzhen Xie, Qiuli Zhu, Junqing Luo, Shengwu Liao, Oudong Xia, Gang Sun

**Affiliations:** 1 Department of Health Management, School of Health Management, Southern Medical University, Guangzhou, 510515 China; 2 Department of Health Policy and Management, Bloomberg School of Public Health, Johns Hopkins University, Baltimore, MD 21205 USA; 3 Department of Health Management, Southern Medical University Nanfang Hospital, Guangzhou, China; 4 Zhujiang Hospital of Southern Medical University, Guangzhou, China

**Keywords:** Elderly population, Health care resource supply and demand, Coupling coordination degree model, Resource allocation

## Abstract

**Object:**

To analyze the trend of the coupling and coordination of the supply and demand of healthcare resources between the elderly population and healthcare resources in China during the period of 2012–2022, to reveal the impact of the growth of the elderly population on the relationship between the supply and demand of healthcare resources, and to put forward suggestions to improve the coupling and coordination between the supply and demand of healthcare resources and the elderly population, in order to cope with the challenges of an aging society.

**Methods:**

By obtaining relevant data from authoritative data sources such as China Statistical Yearbook, Health and Health Statistics Yearbook, and the Chinese government website from 2012 to 2022, we constructed a comprehensive measurement index for the three systems of elderly population, healthcare resource supply, and healthcare resource demand; Using the entropy value method to assign weights to the indicators, combined with the coupling coordination degree model, to reveal the changes of the elderly population change and the supply and demand of medical and health resources; using ArcGIS technology, to study the spatial characteristics of the elderly population change and the supply and demand of medical and health resources.

**Results:**

From 2012 to 2022, the supply and demand of healthcare resources and the variation of the elderly population in China show a continuous growth trend, and the comprehensive development level of the system gradually climbs from a low level to a high level. The fluctuation of coupling degree and coordination degree rises, although the coordination degree has always been lower than the coupling degree, but the distance between the coordination degree and the coupling degree gradually narrows with the passage of time. The coordination degree between population aging and medical and health resources development shows spatial heterogeneity in China, with the eastern region significantly higher than the western region/.

**Conclusions:**

The coupling degree between population aging and healthcare resource supply and demand in China from 2012 to 2022 shows a general upward trend from low coupling to medium-high coupling, but it is worth noting that even though the degree of coupling increases, the degree of coordination is still relatively lagging behind, suggesting that the government and relevant departments need to pay more attention to coordinated allocation and management of healthcare resources. At the same time, the spatial differences in the degree of coordination among provinces suggest that future policymakers should take regional differences into full consideration in policymaking and sustainable development.

## Introduction

Population ageing has become an irreversible global trend, and the problem of ageing is becoming increasingly serious worldwide [1, 2]. China is not only the most populous country in the world, but also one of the fastest aging populations, which poses a serious challenge to a rapidly aging society [3]. According to United Nations standards, a country is considered to be ageing if 10 per cent of its total population is over 60 years of age or 7 per cent of its total population is over 65 years of age [4]. The fifth national census in 2000 showed that the population aged 60 and over had reached 126 million, accounting for 10 per cent of China’s total population, marking the arrival of an ageing society in China [5]. According to China’s seventh national population census in 2020, China’s elderly population over the age of 60 reached 264.02 million, accounting for 18.7% of the total population, an increase of 48.5% in the elderly population and an increase of 10.64% in the proportion of the elderly population as compared with 2010; those over the age of 65 accounted for 13.5% of the total population, an increase of about 4.62% as compared with 2010 [6]. During this decade, life expectancy in China increased from 74.83 years to 77.93 years [7]. It can be concluded that since the beginning of the 21st century, China’s population ageing has accelerated [8]. By the end of 2012, China’s elderly population aged 60 and above had reached 300 million, accounting for more than 20 per cent of the total, marking China’s entry into the stage of moderate ageing. It is projected that by around 2035, the population aged 60 and over will reach 400 million, accounting for more than 30 per cent of the total, marking China’s entry into the stage of heavy ageing. 2050, China’s ageing ratio will reach 44.0 per cent, far higher than the world average of 25.0 per cent [9–11]. It can be seen that the ageing of China’s population will continue to rise steadily.

However, China’s rapid aging leaves insufficient time for the healthcare system to adjust and respond. While the economy is still underdeveloped, China has entered an ageing society ahead of schedule, and the social conditions of “ageing before one is rich” and “ageing before one is ready” have arisen, posing enormous challenges in terms of economic development, old-age security and medical care and health care. Although the technical capacity and quality of healthcare in China has improved significantly over the last decade, according to the 2022 China Health Statistics Bulletin, the number of health institutions, health institution beds, and health technicians is on an increasing trend, and the quality of healthcare services has improved substantially. More notably, however, the growth rate of the number of health institution beds is showing a slowing trend. The reform of public hospitals and the construction of a hierarchical health care system in recent years have provided a strong impetus for the development of geriatric care [12]. To some extent, these reforms have the potential to alleviate the health care needs of an aging population, increase the prioritization of elderly patients, and optimize the use of health care resources for the elderly. However, due to the large size of China’s population, the overall availability of healthcare resources is still insufficient, and it is unclear whether the allocation of healthcare resources is sufficient to meet the needs of an aging society. Overall, the reform of China’s healthcare sector is entering a bottleneck stage, challenging the existing layout of healthcare resource allocation and raising the demand for additional healthcare resources [13, 14].

The World Health Organization has pointed out that the most fundamental problem caused by population ageing is health, and that at the biological level, as the body functions of the elderly continue to deteriorate with age, the demand for health-care resources continues to increase, and at the same time there is a greater need for diversified health-care resources and services. Whether the current supply of medical resources can meet the growing and diversified needs of the elderly has become a hot topic of concern for all sectors of society. By exploring the changing trends of the structure of China’s elderly population in recent years and analyzing the current situation of the supply and demand of healthcare resources, this paper can understand the challenges and impacts of China’s aging process on the healthcare system. By studying the degree of coordination of the coupling tri-system of the elderly population-supply of healthcare resources-demand for healthcare resources, it is not only possible to assess the inadequacy of healthcare resources to cope with the growth of the aging population, but also to provide information for forward-looking policy formulation at the national level [15]. In addition, exploring the spatial and temporal evolution of population aging and healthcare resources can provide targeted guidance for future policy formulation, industrial development and healthcare protection for healthy aging.

## Data and methods

### Data collection

The research period of this paper is 2012–2022, and the data for each indicator come from the China Statistical Yearbook 2011–2023, the official website of the National Bureau of Statistics, the Statistical Bulletin on National Economic and Social Development 2011–2023, the Statistical Bulletin on the Development of China’s Health and Family Planning Programs 2011–2023, the Statistical Yearbook of Health and Wellness, the Statistical Yearbook of Demographics, and the Chinese government website.

This study chooses 2012–2022 as the research time window for several reasons. Firstly, considering the availability and quality of data, this period ensures access to a substantial amount of high-quality national statistical data, reports from the National Health Commission, and data released by other relevant research institutions, which are crucial for the reliability and accuracy of the study. Secondly, we have taken into account the policy background, especially the series of healthcare reform policies introduced by the Chinese government since 2012. Choosing this time window allows us to evaluate the impact and effectiveness of these policies over a relatively long period. Lastly, by selecting a relatively long time window, we aim to capture the long-term trends and changes in the supply and demand of healthcare resources and the aging population. This helps to comprehensively understand and analyze the relationships and interactions between these systems, providing valuable insights for future policy-making.

At the same time, the originally planned data collection period in this study was from 2013 to 2023. However, during the data standardization process, due to the step involving the difference between maximum and minimum values, the lack of data from the previous year (i.e., 2012) could result in some data items for 2012 being zero, thereby affecting the accuracy of the data and the reliability of the research results. To address this issue, we referred to relevant literature and existing research methods and decided to extend the data collection period back to 2011. This adjustment not only avoids anomalies caused by the absence of 2012 data but also ensures the continuity and completeness of data processing, making the time series analysis more reliable. Therefore, extending the data collection period to 2011–2023 provides a more comprehensive baseline, aiding in more accurately capturing trends and changes.

### Data analysis

In order to evaluate the degree of coordination of the elderly population-healthcare resource supply-healthcare resource demand coupling, a comprehensive evaluation of each system is required. First, the selection of indicators in each system is determined based on the combing of existing literature. Second, the data of each indicator selected in each system are standardized and the entropy value method is used to determine the weight of each indicator. Next, the degree of coupling and coordination of the elderly population-healthcare resource supply-healthcare resource demand is determined based on the three-system coupling degree model and the principle of dividing the coupling systems. Finally, the spatial characteristics of the three systems of elderly population-medical and health resources supply-medical and health resources demand are explored by using ARCGIS technology.

### Methodology

#### Entropy value method

The entropy method is initially a thermodynamic concept, known as the information entropy, which is characterized by the degree of difference in the data information to determine the size of the weights, to make an objective evaluation of the system, but also to introduce the concept of “time sequence” to indicate the importance of each stage of each indicator [16–18]. And the objective weights derived from the entropy value method can be used as influencing factors for the comprehensive development level, and the greater the weight, the greater the influence of the indicators on the comprehensive development level [19]. It is also an important step in the measurement of the coupling harmonization index. The calculation process is as follows:

The indicator system for elderly population—healthcare resource supply—healthcare resource demand includes three subsystems. Each of these systems, in turn, consists of a number of subsystems, under which a number of evaluation indicators are included. Indicators are not comparable because different indicators do not have the same unit of measurement. Moreover, there are two types of effects of each indicator on the subsystem, namely, positive and negative effects. A positive effect means that the larger the indicator, the better the subsystem. Conversely, a negative effect means that the larger the indicator, the worse the subsystem. Therefore, this paper needs to standardize the various metrics in the subsystem to eliminate the problems of units of measure and positive and negative roles. The standardized formula is [20–22]:

1 $$x'{\rm{ijt}} = \left\{ \matrix{{{{\rm{xijt}} - {\rm{min(xijt)}}} \over {{\rm{max(xijt)}} - {\rm{min(xijt)}}}}{\rm{, Positive\, indicator}} \hfill \cr {{{\rm{max(xijt)}} - {\rm{xijt}}} \over {{\rm{max(xijt)}} - {\rm{min(xijt)}}}}{\rm{,Negative\, Indicator}} \hfill \cr} \right.$$

In the formula: *xijt*denotes the sample value of indicator j in area i in year t, max and min denote the maximum and minimum values of indicator j in area i, respectively; the normalized *x’ijt* takes values between [0,1].

Individual indicators as a proportion of the total

2 $${\rm{Y}}ijt = {{x'ijt} \over {\sum\limits_{i = 1}^m {x'ijt} }}$$

Calculation of information entropy of indicators

3 $$ej = - k\sum\limits_{i = 1}^m {(Yijt \times \ln Yijt)}$$

Let *k* = 1/ln *m*, then we have 0 ≤ *ef* ≤ 1 and Yijt = 0 at that time, let Y yijt × ln Yijt = 0

Calculation of information entropy redundancy

4 $$dj = 1 - ej$$

Determination of indicator weights

5 $$wj = {{dj} \over {\sum\nolimits_{j = 1}^m {dj} }}$$

The combined evaluation scores for the three systems of elderly population - demand for healthcare resources - supply of healthcare resources are calculated as follows:

6 $${\rm{S}}k,it = \sum\limits_{j = 1}^m {wj \times Yijt}$$

*Sk*,it in the formula can be expressed as the combined development score value of the elderly population, demand for health care resources and health care resources supply system for the i area in year t. *wj* is the weight of the indicator in each subsystem.

#### Coupling coordination degree model

After establishing the evaluation index system of elderly population-medical and health resources supply-medical and health resources demand, each index is assigned by entropy value method, and finally the comprehensive evaluation score of each system from 2012 to 2022 is obtained. According to the coupling degree formula and coordination degree formula, the coupling degree and coordination degree between the three systems can be calculated.

The coupling coordination degree model mainly involves the calculation of three indicators: the comprehensive development level(comprehensive evaluation scores) T value for coupling coordination development, the coupling degree C value, and the coupling coordination degree D value. Comprehensive Development Level (Comprehensive evaluation scores)(T) refers to the overall development status of a system in certain key aspects. In this paper, these aspects include the supply of healthcare resources, the demand for healthcare resources, and the changes in the elderly population. The evaluation of the comprehensive development level is generally measured through a series of indicators that reflect the overall development status of various parts of these three subsystems. Coupling Degree (C) reflects the degree of interdependence and interaction between different subsystems within a system. In this paper, the coupling degree is used to describe the extent of the connection between the subsystems of healthcare resource supply, healthcare resource demand, and changes in the elderly population. A high coupling degree means that these subsystems are closely linked and will influence each other when changes occur. The coupling degree helps to understand the interconnectedness of the subsystems and indicates whether they are operating in a coordinated manner or there are conflicts. Coordination Degree (D) represents the effectiveness and coherence of cooperation between different subsystems within a system. A high coordination degree means that the subsystems can work well together to jointly promote the overall development of the system. The coordination degree reflects whether the subsystems of healthcare resource supply, healthcare resource demand, and changes in the elderly population can develop synchronously, thereby avoiding negative impacts on other dimensions caused by the development of a single dimension.

The concept of coupling was originally discovered in physics and is defined as a phenomenon in which two or more systems interact with each other [23]. This paper borrows the concept and model of capacity coupling from physics to calculate the coupling degree of elderly population-healthcare resource demand-healthcare resource supply, and the coupling degree (C) is calculated as follows [24]: U1, U2 and U3 are used to represent the comprehensive scores of the elderly population sub-system, the healthcare resource demand sub-system and the healthcare resource supply sub-system, respectively. subsystems, then the coupling degree formula of the elderly population-healthcare resource demand-healthcare resource supply three systems is shown in Eq. (7):

7 $$C = {\left[ {{{{\rm{U}}1 \times U2 \times U3} \over {{{\left( {{{U1 + U2 + U3} \over 3}} \right)}^3}}}} \right]^{{1 \over 3}}}$$

The coupling degree C in the three-system coupling degree formula (7) takes values between [0, 1]. Similarly, the coupling degree reaches the maximum value when and only when U1 = U2 = U3.1 The larger the value of C, the higher the degree of association of the subsystems and the stronger the interaction.Referring to the research of related scholars, the division standard of the coupling degree is determined as shown in Table 1.

**Table 1:** Criteria for classifying coupling

Coupling C interval	Coupling degree
0	Stages of Disorderly Development
[0, 0.3)	Low-level coupling development stage
[0.3, 0.5)	Antagonistic developmental stage
[0.5, 0.8)	Break-in development stage
[0.8, 1)	High-level coupling development stage
1	Benign resonance stage

After calculating the coupling degree, the total comprehensive evaluation score of the elderly population, demand for health care resources, and supply of health care resources for the three systems can be calculated, as shown in Eq. (8):

8 $${\rm{T}} = \beta 1{\rm{U}}1 + \beta 2{\rm{U}}2 + \beta 3{\rm{U}}3$$

T is the total development score of the three systems, which is used to reflect the complementary relationship among the subsystems. Where β is a coefficient to be determined, which is used as the weight of the comprehensive evaluation score. The weights here are not the objective entropy value assignment method mentioned above, but are set artificially. Here, because the three systems can compensate each other, the size of the weight reflects the importance of the three systems, and this paper considers that the elderly population, the demand for medical and health resources, and the utilization of medical and health resources are all equally important, so all three coefficients are set to 1/3.

After calculating the degree of coupling of the three systems and the comprehensive development level, this paper chooses the objective quartile method, and divides the score level from low to high into four stages: low level, medium level, high level and high level stage.

Although the coupling degree can show the strength of the roles between the systems, it cannot show the overall coordination of the systems. The degree of coordination can better judge the degree of coupling coordination between the three systems. Therefore, this paper constructs a suitable coupling coordination degree model with the following formula:

9 $$D = \sqrt {C \times T}$$

D in Eq. (9) represents the degree of coordination, and the larger the value of D, the higher the overall development level of the subsystem and the degree of coordination between systems. Referring to existing studies, the coupling coordination development stage and development type can be divided [25], see Table 2.

**Table 2:** Criteria for classifying coupling coordination degree

Interval of D-values for coupling coordination	Degree of coupling coordination	Type of coupling coordination
<0.20	Serious dissonance	Dysfunctional recession category
[0.20,0.40)	Mild disorder	
[0.40,0.60)	General coordination	Overdevelopment category
[0.60,0.80)	Moderate coordination	Coordinated development category
[0.80,1]	Highly coordinated	

## Results

### Indicator evaluation system for elderly population-healthcare resource supply-healthcare resource demand

Based on the characteristics of the coupling and coordination system of elderly population, healthcare resource supply and healthcare resource demand, following the principles of scientificity, wholeness, hierarchy and data availability, and referring to the papers on the analysis of coupling and coordination analysis, this paper lists the comprehensive measurement indicators of the three systems of elderly population, healthcare resource supply and healthcare resource supply, which are shown in Table 3. In this paper, we set out the comprehensive measurement indexes of the three systems of elderly population, medical resource demand and medical resource supply, which are shown in Table 3, and the rightmost column of Table 3 shows the average weights of each index in the past years, and for the calculation of the weights, please refer to the Sect. 2.3.1, “Entropy method of assigning weights”.

**Table 3:** Comprehensive evaluation indicators for 3 systems of Elderly Population, demand for Healthcare resources and Supply of Healthcare resources

System	Subsystem	Evaluation indicators	Unit	Positive or Negative	Average weighting
Elderly population	Size of the population	Number of persons aged ≥ 60 years	10,000 people	Positive	0.1154
		Number of persons aged ≥ 65 years	10,000 people	Positive	0.1605
	Densitization of the population	Density of older persons aged ≥ 60 years	people/km^2^	Positive	0.1153
		Density of older persons aged ≥ 65 years	people/km^2^	Positive	0.1605
	demographic structure	Proportion of population aged ≥ 60 years	%	Positive	0.1125
		Proportion of population aged ≥ 65 years	%	Positive	0.1623
		Dependency ratio of the elderly population aged 65 and over	%	Positive	0.1734
Demand of medical and health resources	Medical services utilization rate	Hospital bed occupancy rate	%	Positive	0.1649
		Daily Visits /Per Doctor	person-time	Positive	0.1249
		Daily Inpatients /Per Doctor	day	Positive	0.1485
		Number of visits per inhabitant per year	time	Positive	0.1279
	Medical costs	Total medical expenditures	hundred million RMB	Negative	0.0575
		Per capita medical costs	RMB	Negative	0.0562
		Percentage of out-of-pocket medical expenses	%	Negative	0.3201
Supply of medical and health resources	Hospital	Number of hospitals	piece	Positive	0.1355
		Number of hospital beds	10,000 beds	Positive	0.1271
		Number of hospital health technicians	10,000 people	Positive	0.1333
	Primary health care institution	Number of primary health care institutions	piece	Positive	0.2287
		Number of beds in primary health care	10,000 beds	Positive	0.1704
		Number of primary health care technicians	10,000 people	Positive	0.2049

Among the indicators that reflect the older population are population size, population density and population structure. The most direct indicator that can measure the size of the older population is the total older population, that is, the total number of inhabitants aged 60 years and over in a given area or in the country as a whole. At the same time, population density is an important indicator of the distribution and concentration of older persons in a country or region. The higher the proportion of the population aged 60 years or over and the proportion of the population aged 65 years or over, the higher the degree of population ageing in general. Population structure is also a key indicator of the characteristics of the elderly population. The degree of population aging in a country or region can be effectively measured by looking at the population structure. The degree of ageing in the population structure can be reflected by the number of persons in the 60-and-over and 65-and-over age groups as a proportion of the total population. When the proportion of the population aged 60 years and over or 65 years and over is high, it usually indicates a higher level of population ageing in the country or area.

Two subsystems, medical service utilization rate and medical cost, have been chosen for the medical resource demand indicators, which are essential and important indicators for measuring the demand for medical resources. The healthcare service utilization rate can reflect the load of the community or the entire healthcare system and help assess the efficiency and trend of healthcare resource use. Healthcare costs, on the other hand, are directly related to the financial burden on patients and the cost of healthcare resources, and are an important basis for assessing the balance between demand and supply of healthcare resources. If these indicators are too high, then they will also inhibit people’s demand for health care to some extent.

Two systems, hospitals and primary health care institutions, have been selected as indicators of the supply of health care resources. The number of medical institutions and the number of medical and health-care technicians have a direct impact on the supply of medical and health-care resources, while the number of beds in medical and health-care institutions reflects the scale and capacity of the inpatient treatment services that medical institutions can provide. The number of beds is directly related to whether or not patients can receive timely hospitalization when they seek treatment, and affects the accessibility and satisfaction of medical services. Therefore, the number of beds in healthcare institutions is also one of the important indicators for assessing whether the supply of healthcare resources is sufficiently abundant. Taking into account such indicators as the number of hospitals, the number of medical and health-care technicians and the number of beds in medical and health-care institutions, it is possible to assess the supply of medical resources in a more comprehensive manner and to provide a scientific basis for the planning of the medical and health-care system and the allocation of resources.

### Trends and evolution of supply and demand of population ageing and healthcare resources, 2012–2022

As shown in Table 4, the structure of China’s elderly population has changed significantly over the past decade, showing a clear trend of population aging. From 2012 to 2022, the number of elderly people aged ≥ 60 and ≥ 65 continued to grow, from 193.9 million and 127.14 million to 28.04 million and 209.78 million, an increase of 44.31 million and 82.77 million, respectively, and the density of the elderly population increased accordingly, reflecting the trend of a year-on-year increase in the proportion of the elderly population in the total population. In addition, the proportion of elderly people aged ≥ 60 and ≥ 65 years has also increased, from 14.3% and 9.4–19.8% and 14.9%, an increase of 5.5% points and 5.5% points, which further reinforces the reality of an ageing population. As the proportion of the elderly population increases, the dependency ratio of the elderly population aged 65 years and over also shows a year-on-year trend of increase, rising from 12.7 to 21.8, an increase of 9.1% points, which implies that the elderly population has an increasing need for the old-age support of the working population, as well as an increasing demand for social resources such as social services for the aged and medical and health-care resources. These figures therefore highlight the profound changes in China’s social structure, forcing the Government and all sectors of society to adopt effective policies and measures to meet the challenges posed by an ageing society.

**Table 4:** Elderly Population indicators 2012–2022

Year	Number of persons aged ≥ 60 years(10,000 people)	Number of persons aged ≥ 65 years(10,000 people)	Density of older persons aged ≥ 60 years(people/km^2^)	Density of older persons aged ≥ 65 years(people/km^2^)	Proportion of population aged ≥ 60 years(%)	Proportion of population aged ≥ 65 years(%)	Dependency ratio of the elderly population aged 65 and over(%)
2012	19,390	12,714	20.21	13.24	14.3	9.4	12.7
2013	20,243	13,161	21.09	13.71	14.9	9.7	13.1
2014	21,242	13,755	22.13	14.33	15.5	10.1	13.7
2015	22,200	14,386	23.13	14.96	16.1	10.5	14.3
2016	23,086	15,003	24.05	15.63	16.7	10.8	15.0
2017	24,090	15,831	25.10	16.49	17.3	11.4	15.9
2018	24,949	16,658	25.99	17.35	17.9	11.9	16.8
2019	25,388	17,603	26.45	18.34	18.1	12.6	17.8
2020	26,402	19,064	27.50	19.86	18.7	13.5	19.7
2021	26,736	20,056	27.85	20.89	18.9	14.2	20.8
2022	28,004	20,978	29.17	21.85	19.8	14.9	21.8

As shown in Table 5, China’s healthcare system has experienced significant growth over the past decade. From 2012 to 2022, the number of hospitals increased from 23,170 to 36,976, an increase of nearly 59.5%. The number of actual hospital beds increased from 4,161,500 to 7,662,900, an increase of nearly 84.1%. The number of hospital health technicians increased from 4,058,000 to 7,353,000, an increase of nearly 81.2%. At the same time, the number of primary health care organizations increased from 912,620 to 979,768, a 7.3% increase. The number of beds in primary health-care organizations increased from 1,324,300 to 1,751,100, an increase of 31.8 per cent. The number of primary health care technicians increased from 2,052,000 to 3,450,000, an increase of 68.2%. The upward trend reflects the Government’s emphasis and investment in the health-care system. The increase in the number of hospitals and beds means that more patients have access to timely medical services and treatment, while the increase in the number of health-care technicians has improved the quality and efficiency of medical services. In addition, the increase in the number of primary health-care institutions has made medical resources more widely available and decentralized, helping to meet the health needs of grass-roots residents. These figures highlight the strong momentum of China’s medical and health care sector, which has continued to grow over the past decade, providing a reliable foundation and support for safeguarding the health of the entire population.

**Table 5:** Indicators of Healthcare Resource Supply, 2012–2022

Year	Number of hospitals(size)	Number of hospital beds(10,000 beds)	Number of hospital health technicians(10,000 people)	Number of primary health care institutions(size)	Number of beds in primary health care(10,000 beds)	Number of primary health care technicians(10,000 people)
2012	23,170	416.15	405.8	912,620	132.43	205.2
2013	24,709	457.86	442.5	915,368	134.99	213.8
2014	25,860	496.12	474.2	917,335	138.12	217.2
2015	27,587	533.06	507.1	920,770	141.38	225.8
2016	29,140	568.89	541.5	926,518	144.19	235.4
2017	31,056	612.05	578.8	933,024	152.85	250.5
2018	33,009	651.97	612.9	943,639	158.36	268.3
2019	34,354	686.65	648.7	954,390	163.11	292.1
2020	35,394	713.12	677.5	970,036	164.94	312.4
2021	36,570	741.42	711.5	977,790	169.98	330.2
2022	36,976	766.29	735.3	979,768	175.11	345.0

As shown in Table 6, over the past decade, China’s healthcare sector has experienced booming development and the demand for healthcare resources has continued to grow. The data show that from 2012 to 2022, China’s total healthcare costs will grow from 2,811.9 billion yuan to 8,532.7 billion yuan, and per capita healthcare costs will increase from 2,068.76 yuan to 6,044.09 yuan, reflecting the rising demand for healthcare services in terms of both quantity and quality. In terms of the utilization of medical resources, while the rate of bed utilization has remained relatively stable, the average number of inpatient bed-days and the number of consultations per medical practitioner have remained at a high level, indicating that the demand for medical services is still very strong. At the same time, the number of outpatient visits per capita per year has been rising year by year, indicating that people’s attention to and demand for health is increasing. Overall, China has made remarkable achievements in the development of its medical and health care sector, but also faces the challenge of inadequate supply of medical resources.

**Table 6:** Indicators of demand for health resources, 2012–2022

Year	Total medical expenditures(billion)	Per capita medical costs(Chinese yuan)	Percentage of out-of-pocket medical expenses(%)	Hospital bed occupancy rate(%)	Daily Inpatients /Per Doctor(day)	Daily Visits /Per Doctor(person-time)	Number of visits per inhabitant per year(time)
2012	28119.00	2068.76	44.0	82.80	2.6	7.20	5.1
2013	31668.95	2316.23	33.9	82.40	2.6	7.30	5.4
2014	35312.40	2565.45	31.99	81.60	2.6	7.50	5.6
2015	40974.64	2962.18	29.27	79.50	2.6	7.30	5.6
2016	46344.88	3328.61	28.78	79.80	2.6	7.30	5.7
2017	52598.28	3756.72	28.77	79.70	2.6	7.10	5.9
2018	59121.91	4206.74	43.60	78.80	2.5	7.00	6
2019	65841.39	4669.34	28.36	78.02	2.5	7.10	6.24
2020	72175.00	5112.34	27.65	67.65	2.2	5.90	5.5
2021	76844.99	5439.97	27.60	69.28	2.2	6.50	6
2022	85327.49	6044.09	26.89	66.10	2.1	6.20	6

### Degree of coordination of the overall coupling between population aging and the supply and demand of health care resources

**Fig. 1:**
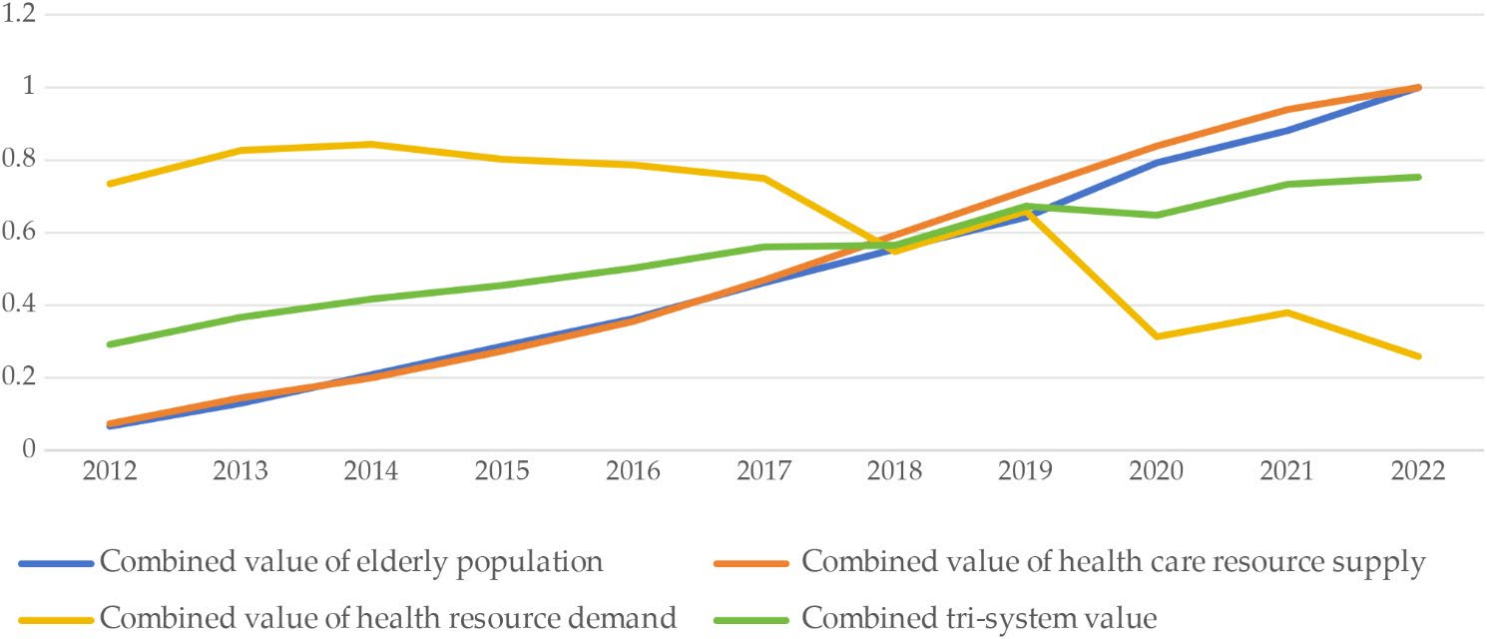
Combined value of the elderly population-supply of health care resources-demand for health care resources system

The classification of comprehensive evaluation scores at the national level for China’s elderly population—healthcare resource supply—healthcare resource demand from 2012 to 2022, derived using the objective quartile method, is shown in Table 7. The trend of the comprehensive scores for each subsystem (elderly population, healthcare resource supply, and healthcare resource demand) is shown in Fig. 1. The combined value of the elderly population system, the healthcare resource supply system and the healthcare resource demand system has a clear trend in the past decade. The composite value of the elderly population system shows an increasing trend year by year, from 0.0665 in 2012 to 1 in 2022, which shows that the number of the elderly population is increasing year by year, and there is a clear trend of aging. The composite value of the health care resource supply system continues to increase between 2012 and 2019, but declines in 2020 and 2021, reflecting an under- or uneven supply of health care resources in both years. Whereas the combined value of the healthcare resource demand system fluctuates throughout the time period, it generally shows an increasing trend from year to year, highlighting the growing demand for healthcare services. T, as a composite evaluation of the three systems of the elderly population, healthcare resource supply and healthcare resource demand, has shown a positive upward trend from 20,122, indicating a gradual improvement in the overall healthcare system in meeting the needs of the elderly population. However, it is important to note that although the composite value T shows an increasing trend overall, there has been a decline in some years, especially in the healthcare resource supply system.

**Table 7:** Elderly Population-Healthcare Resource Supply-Healthcare Resource demand comprehensive evaluation score classification

Year	2012	2013	2014	2015	2016	2017	2018	2019	2020	2021	2022
**Classification**	Low level	Low level	Low level	Low level	Medium level	Relatively high level	Medium level	High level	Relatively high level	High level	High level

**Fig. 2:**
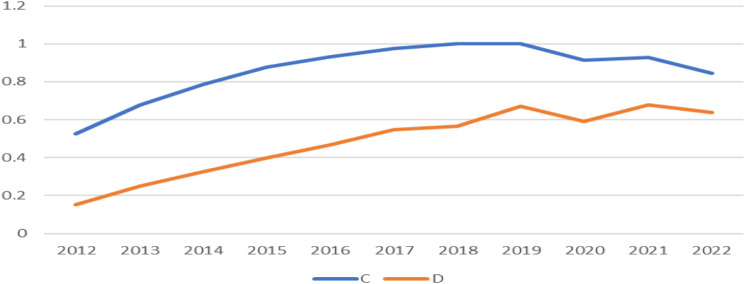
The average Coupling Degree (C Value) and Coordination Degree (D Value) of the three systems from 2012 to 2022

The harmonization of supply and demand of China’s elderly population and healthcare resources at the national level for 2012–2022 was calculated using CCDM. The overall change in the degree of coordination of the coupling between the elderly population and the supply and demand of healthcare resources, using the C and D values as indicators, is visualized in Fig. 2. Figure 2 illustrates the overall change in this coupling and coordination degree, clearly presenting the evolution of the relationship between the elderly population and the supply and demand of healthcare resources over time. Based on the overall coupling coordination analysis, several key points can be obtained. The degree of coupling coordination at the national level shows an overall upward trend during the study period, with the value of C increasing from 0.525 in 2012 to 0.846 in 2022, while the value of D increases from 0.153 in 2012 to 0.637 in 2022.This implies that the degree of coupling between the supply and demand of the elderly population and healthcare resources gradually increases from a low coupling during the period from 2012 to 2022 increasing to a high coupling level. This result reflects the gradual harmonization of the relationship between the growth of the elderly population and the supply and demand of healthcare resources. As the elderly population increases, the demand for healthcare resources also increases, while the supply of healthcare resources also rises accordingly, thus realizing an increase in the degree of coupling.

### Spatial characterization of coupling coordination degrees

**Fig. 3:**
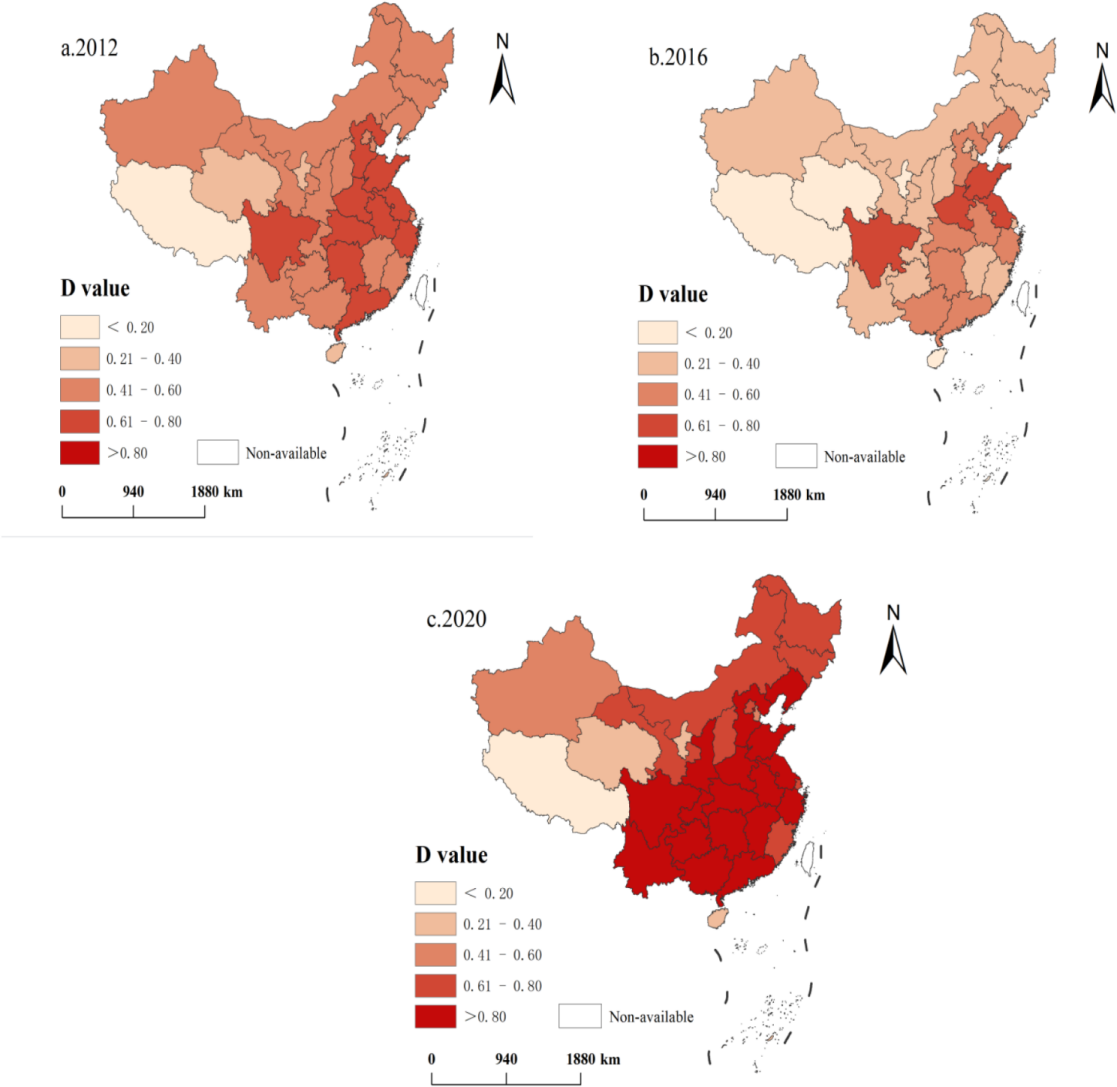
Spatial distribution of the degree of coordination (D-value) of the three-system coupling(4-year interval)

To analyze the evolution of the coupling coordination level of population aging and economic growth at the provincial level in China, the years 2012, 2016, and 2020 were used as the cross-sectional years during the study period. Then the coupling coordination levels at the provincial level in China are classified and visualized, and Fig. 3 shows the spatial distribution of the coupling coordination level values of 31 provincial units in mainland China in 2012, 2016, and 2020.

Most of the regions with higher coupling harmonization values in 2012 belonged to the central and southeastern coastal regions, with the specific provinces being Shandong, Henan, Hubei, Hunan, Guangdong, Sichuan, Jiangsu, Zhejiang, as well as Anhui and Hebei. Guangdong, Zhejiang, and Jiangsu are leading in China’s economic development and have high levels of healthcare resource supply, and these regions are relatively rich in healthcare resources, which can better meet the healthcare needs of the elderly population; compared to some economically developed regions, the distribution of the elderly population in the central region may be relatively balanced, with no concentration of the elderly population like that of the first-tier cities, making the better match between the supply and demand of healthcare resources. As a result, these regions have the highest coupling coordination values in the country. However, the regions with low coupling coordination are the three western regions of Tibet, Qinghai and Ningxia, as well as the southern region of Hainan. Due to the geographical conditions and the level of economic development, the relative lack of health care resources in these regions, the relatively high proportion of elderly population with low level of health care services has led to a prominent contradiction between supply and demand, coupling with the relative backwardness of the regional economy, a series of factors have led to the low value of the coupling coordination of the three systems, namely, the elderly population, health care resource supply and health care resource demand. A series of factors have led to the low value of the coupling coordination degree of the three systems of elderly population-medical and health resources supply-medical and health resources demand in these regions. The other provinces have a moderate degree of coordination. The scores of the three systems in these provinces are relatively balanced, but there are shortcomings in some regions, so the coupling degree is at the medium level.

The spatial distribution of the coupling coordination degree in 2016 shows a large change compared with 2012. Except for the four provinces of Jiangsu, Shandong, Henan, and Sichuan, which maintained a higher coupling coordination degree value of the three systems of elderly population-healthcare resource supply-healthcare resource demand, the six regions with higher coupling coordination degree values, Hebei, Zhejiang, Anhui, Hunan, Hubei, and Guangdong, all declined to medium coupling areas. The two provinces of Guangxi and Liaoning remain in the ranks of moderately coupling regions, while the rest of the provinces decline to low coupling coordination regions.

From the viewpoint of the spatial distribution of the coupling degree of coordination in 2020, compared with 2016, the overall coupling degree of coordination of all provinces in the country shows a favorable development trend, and the overall coupling degree of coordination exceeds that of 2012 and moves upward by a large margin. Except for Qinghai, Ningxia, Tibet and Hainan, which are still low-coupling-coordination areas, and Xinjiang and Tianjin, which are in medium-coupling-coordination areas, the rest of the regions have all risen to the ranks of high or high-coupling-coordination areas.

In general, a clear difference in D-values between eastern and western China can be seen. Eastern provinces such as Jiangsu, Shanghai, Zhejiang, Shandong, Fujian, and Beijing always have high D-values (> 0.6), indicating medium or high coupling. And the D-values of western provinces such as Tibet, Qinghai, Ningxia, and Gansu are the lowest in China, indicating low or medium decoupling. Second, compared with 2016, the D-values of most provinces such as Shaanxi and Guizhou show an increasing trend. In the process of population aging and healthcare resource gaming, the degree of coupling and coordination between these two systems shows obvious regional differentiation.

## Discussion

### Challenges of aging and the balance of supply and demand of healthcare resources under the comprehensive development level perspective

Over the past decade, the rising level of socio-economic development has had a profound impact on the three systems of the elderly population, the supply of healthcare resources and the demand for healthcare resources. Using the Comprehensive Development Score as a measure, as shown in Fig. 1, we can find a continuous growth trend from 2012 to 2022, with the Comprehensive Development Score gradually climbing from a low level to a high level, reflecting a significant increase in the overall level of social and medical development. With the rapid development of society and economy, the elderly population is also showing a trend of growth in number and proportion year by year. This means that the demand for medical and health resources continues to climb, and the elderly population’s need for medical services is becoming more and more urgent. In particular, as people’s income level rises and their health awareness increases, their desire for quality medical services is also growing.

With regard to the supply of healthcare resources, between 2012 and 2022, the Chinese government and all sectors of society have increased investment in the healthcare sector, the coverage of healthcare resources has been expanding, the quality of healthcare resources has improved markedly, the level of healthcare facilities and technology has steadily risen, the efficiency and accessibility of the overall healthcare system has been improving, and the improvements in the healthcare system better meet the needs for healthcare resources of the elderly population and society as a whole, and the level of comprehensive development has been rising. Improvements in the health-care system have better met the needs of the elderly population and society as a whole for health-care resources, and the level of comprehensive development has continued to rise. Thus, the elderly population, the supply of health-care resources and the demand for health-care resources constitute an interrelated system.

### Coupling and coordinated relationship between the supply and demand of health care resources and the growth of the elderly population

The coupling degree and coordination degree are important indicators of the relationship between the growth of the elderly population and the supply and demand of healthcare resources. From Fig. 2, the average values of the coupling degree and coordination degree of the three systems of the elderly population-medical and healthcare resource supply-medical and healthcare resource demand in China show a fluctuating and increasing trend from 2012 to 2022. As shown in Table 1, the coupling degree level shifted from a grinding development stage to a high level of coupling development stage; as shown in Table 2, the degree of coupling coordination continuously shifted from dysfunction to a high stage of coordination, and the coupling coordination category stabilized from a dysfunctional declining category to a coordinated development category via a transitional development category. From the data and trends obtained, the increase in the degree of coupling and coordination reflects the gradual realization of the impact of the growth of the elderly population on health care resources and the progress made by the relevant sectors in resource allocation and demand management. First, the year-on-year increase in the degree of coupling indicates an increasingly close link between the growth of the elderly population and the supply of and demand for medical and health resources. This means that the increase in the elderly population has had a more significant impact on the demand for medical and health resources, requiring the Government and relevant departments to pay more attention to the health needs of the elderly population and to ensure that medical and health resources can effectively meet the demand. Secondly, the increase in the degree of coordination shows that the relevant sectors have made some achievements in resource allocation and demand management over the past decade. However, despite the increase in coordination, a certain degree of inadequacy remains. From Fig. 2, the degree of coordination has been below the degree of coupling, on the one hand, which indicates that the degree of coordination between the supply of and demand for health care resources has not reached a level corresponding to the growth of the elderly population throughout the observation period. Even though the coupling degree has increased, the degree of coordination is still relatively lagging behind, which means that the growth and adjustment of health care resources cannot keep up with the changes in health care demand brought about by the growth of the elderly population. This situation may lead to insufficient supply of healthcare resources in some areas or groups, affecting the quality and efficiency of the elderly population’s access to healthcare services. On the other hand, it also suggests the need for the Government and relevant departments to pay more attention to the coordinated allocation and management of health-care resources. With the continuing trend of growth in the elderly population, the focus has been on the distribution of demand for medical and health resources, and the deployment and management of resources has been strengthened to ensure that the medical and health needs of the elderly population are met in a more balanced and adequate manner, and that medical and health services are able to effectively cover all parts of the country and meet the health needs of the elderly population.

Overall, in the relationship between the supply and demand of health care resources and the growth of the elderly population, the degree of coordination has remained below the degree of coupling, but the distance between the two has gradually narrowed, indicating an encouraging trend: the balanced relationship between the supply of health care resources and the growth of the elderly population is gradually strengthening and improving. During the period 2012–2022, the distance between coordination and coupling has gradually narrowed over time, which means that China’s relevant authorities have begun to take proactive measures to improve the level of healthcare resource provision to better cater to the needs of an aging society.During the period 2012–2022, the Chinese government and the relevant organizations have been increasing investment in healthcare resources, optimizing resource allocation, strengthening service coverage, and improving the quality and efficiency of healthcare services. Over the past decade, the Government and relevant authorities have worked to improve the coordination between the supply of health-care resources and the growth of the elderly population, and have introduced a series of initiatives that have helped to alleviate the shortage of health-care resources and better meet the challenges posed by the growth of the elderly population.

### Spatial characterization of the coupling coordination of supply and demand of healthcare resources and growth of the elderly population

Based on the research results of CCDM, it has been found that there are significant differences in the degree of coupling coordination between the elderly population and the supply and demand of medical resources at both the national and provincial levels in China. Provinces in the western regions such as Tibet, Qinghai, and Ningxia have lower D values, reflecting a low coupling state among the three systems of elderly population, the supply of medical and health resources, and the demand for medical and health resources. This indicates that there is significant spatial differentiation in the degree of coupling coordination between population aging and the development of medical and health resources in China. Therefore, in future policy making and sustainable development, policymakers should fully consider regional differences in various areas and provide corresponding policy support to these regions.

Furthermore, spatial distribution analysis from Fig. 3 reveals that directly-administered municipalities and relatively underdeveloped areas exhibit similar levels of coupling coordination. However, the influencing factors vary significantly between these regions. Directly-administered municipalities are more affected by internal challenges stemming from uneven resource allocation, whereas relatively underdeveloped areas are influenced more by differences in policy formulation and implementation. Therefore, in addressing the mismatch between supply and demand of medical and health resources in directly-administered municipalities and underdeveloped regions, policymakers need to adopt differentiated policy measures tailored to the characteristics of each region. This approach aims to achieve more precise and effective resource allocation and demand fulfillment.

In addition, the results of the previous study show that the degree and differences of China’s aging population continue to increase, and the demand for healthcare resources continues to expand, but the reform of China’s healthcare system is entering a deep-water zone and a zone of attack, which means that the supply of healthcare services has reached a bottleneck stage, so policymakers must grasp the scale of the next stage of the reform of the healthcare system, to prevent the emergence of a situation similar to that of the 2016 Coupling and Coordination degree of regression to ensure the continued supply and improvement of healthcare services.

## Conclusion

Based on the time-series evolution of China’s population aging and healthcare resource indicators from 2012 to 2022, this paper adopts the coupling coordination degree model to firstly evaluate the degree of coupling coordination between China’s population aging and healthcare resource supply and demand at the national level, and elaborates that the period from 2002 to 2020 shows a general upward trend from low coupling to medium-high coupling. to medium-high coupling, but it is worth noting that the degree of coordination has been at the bottom of the coupling degree, and even though the coupling degree has increased, the degree of coordination is still relatively lagging behind, which means that the growth and adjustment of healthcare resources cannot keep up with the changes in healthcare demand brought about by the growth of the elderly population, suggesting the need for the government and the relevant departments to pay more attention to the coordinated allocation and management of healthcare resources. Secondly, the spatial characteristics of the coupling and coordination values of each province were evaluated at the provincial level, and the results of the study revealed the spatial differences in the degree of coupling and coordination among provinces, showing that the eastern coastal region is higher than that of the western region, which suggests that future policymakers should take regional differences into full consideration in policymaking and sustainable development, and give policy preferences and support to these regions.

## Data Availability

No datasets were generated or analysed during the current study.

## Abbreviations

CCDM: Coupling Coordination Degree Model
